# The Handling and Sampling of Radical Cystectomy Specimens: A Standardized Approach for Pathological Evaluation

**DOI:** 10.3390/mps8020035

**Published:** 2025-04-05

**Authors:** Francesca Sanguedolce, Angelo Cormio, Magda Zanelli, Maurizio Zizzo, Andrea Palicelli, Alessandra Filosa, Ugo Giovanni Falagario, Andrea Benedetto Galosi, Luigi Cormio, Giuseppe Carrieri, Roberta Mazzucchelli

**Affiliations:** 1 Pathology Unit, Policlinico Foggia, University of Foggia, 71122 Foggia, Italy; francesca.sanguedolce@unifg.it; 2 Department of Urology, Azienda Ospedaliero-Universitaria Ospedali Riuniti di Ancona, Università Politecnica Delle Marche, Via Conca 71, 60126 Ancona, Italy; a.b.galosi@univpm.it; 3 Pathology Unit, Azienda USL-IRCCS di Reggio Emilia, 42123 Reggio Emilia, Italy; magda.zanelli@ausl.re.it (M.Z.); andrea.palicelli@ausl.re.it (A.P.); 4 Surgical Oncology Unit, Azienda USL-IRCCS di Reggio Emilia, 42123 Reggio Emilia, Italy; maurizio.zizzo@ausl.re.it; 5 Section of Pathological Anatomy, Department of Biomedical Sciences and Public Health, United Hospitals, Università Politecnica delle Marche, 60126 Ancona, Italy; a.filosa@univpm.it (A.F.); r.mazzucchelli@univpm.it (R.M.); 6 Department of Urology and Renal Transplantation, Policlinico Foggia, University of Foggia, 71122 Foggia, Italy; ugo.falagario@unifg.it (U.G.F.); luigi.cormio@unifg.it (L.C.); giuseppe.carrieri@unifg.it (G.C.); 7 Department of Urology, Bonomo Teaching Hospital, 76123 Andria, Italy

**Keywords:** radical cystectomy, bladder cancer, histopathological evaluation, specimens, standardized protocols

## Abstract

An accurate histopathological evaluation of radical cystectomy (RC) specimens is crucial for optimal tumor staging, prognosis, and therapeutic decision making. The increasing demand for precision medicine and multidisciplinary oncological management emphasizes the necessity for standardized protocols in the handling and sampling of bladder cancer specimens. The effective processing of RC specimens begins with the integration of clinical and anamnestic data, along with appropriate formalin fixation methods to meet diagnostic needs. The pathologist must meticulously document the macroscopic characteristics and dimensions of the surgical specimen, especially in post-neoadjuvant chemotherapy (post-NAC) cases where the primary tumor may not be macroscopically visible. Sampling strategies should ensure a comprehensive assessment of the primary tumor and any extra-organ or metastatic involvement. Despite international guidelines, variability in pathology practices persists, particularly concerning prostate sampling in RC and the use of frozen sections for margin assessment. Addressing these challenges necessitates a consensus-driven, standardized approach to improve the reproducibility and quality of histopathological data. By addressing gaps in current pathology practices, this review advocates for uniform protocols that enhance diagnostic accuracy, ultimately improving patient care and clinical decision making.

## 1. Introduction

Accurate histopathological reporting starts with a careful macroscopic examination of pathological specimens, ensuring a thorough diagnostic and prognostic assessment. The demands of precision medicine and multidisciplinary care necessitate an optimal balance in surgical specimen handling. High-quality sections must be obtained to effectively represent the tumor (or its absence after prior treatment) and document all microscopically identifiable diagnostic and prognostic parameters. Additionally, timing and technical resources should be optimized within a workflow that accommodates both pathology laboratory efficiency and the clinical management of bladder cancer patients.

Although there are international guidelines such as those developed by the College of American Pathologists (CAP), the International Collaboration on Cancer Reporting (ICCR), the British Association of Urological Pathologists (BAUP), and The Royal College of Pathologists of Australasia (RCPA), a full standardization about the methods for sampling a cystectomy specimen has not yet been achieved. In fact, all these international associations provide recommendations solely on the features to be included in pathological reports for cystectomies, ensuring their usefulness in clinical practice. So, significant variability persists in clinical practice regarding the sampling of surgical specimens due to differences in local, national, and institutional protocols, as well as variations in their implementation across individual laboratories. Resource availability, such as the presence of pathology assistants or dedicated uropathologists, further influences these disparities. Standardization is widely recognized as essential for enhancing process quality and clinical outcomes.

This review, rooted in the collaboration between uropathologists and urologists and informed by the latest literature, aims to refine current practices while offering insights for future methodological advancements. Any protocol or guideline must be tailored to the specific needs of each laboratory and communicated with the clinical team to ensure its seamless integration. Ultimately, proper specimen handling and sampling guarantee the acquisition of comprehensive diagnostic and prognostic data, allowing clinicians to make well-informed treatment decisions.

## 2. Before Starting

**Figure 1:**
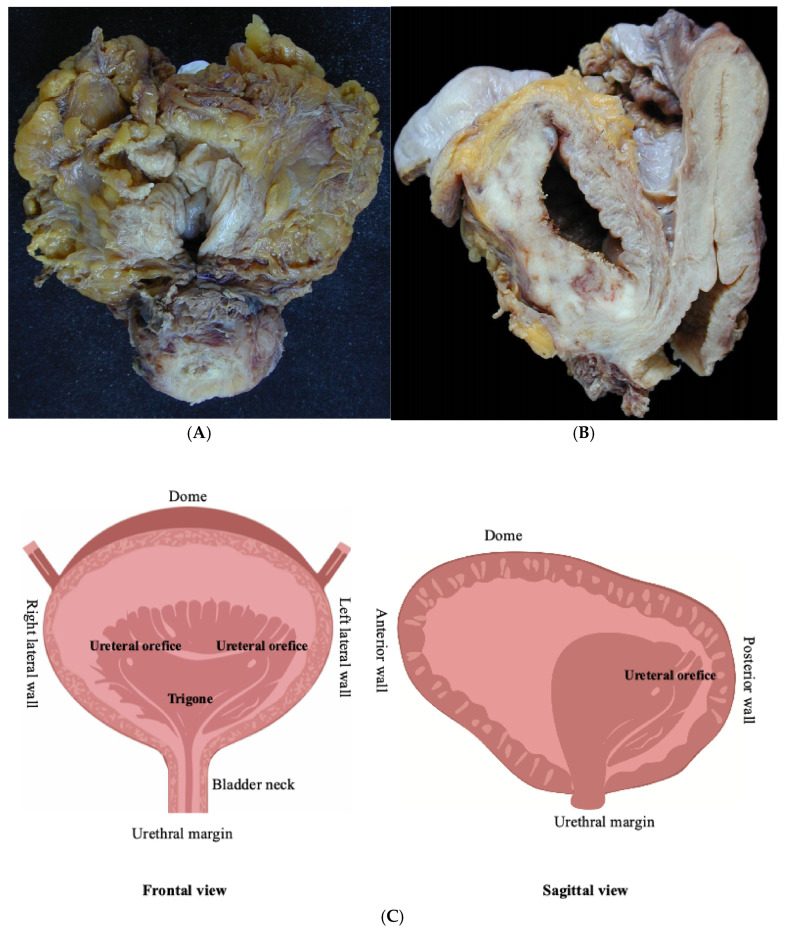
Standard surgical specimens: (**A**) radical cystoprostatectomy in a man and (**B**) radical cystectomy and hysterectomy in a woman, (**C**) schematic drawing of the anatomical regions of urinary bladder.

The standard RC specimen usually includes the distal portion of the ureters, in direct continuity with the ureteral orifices. Unless previously removed, the specimen also includes the prostate and seminal vesicles in men, and the urethra, adjacent vagina (vaginal vault), and uterus in women. The orientation of the RC surgical specimen is facilitated by the presence of organs adjacent to the bladder and by the assessment of the peritoneal covering (Figure 1A,B).

Essential prerequisites for the proper handling of surgical specimens by the pathologist include the following:

- The correct identification of the material contained in each container sent to the pathology laboratory, ensured by the proper labeling of the containers. The contents must be accurately documented in the accompanying request form, whether paper-based or digital.
- The communication of all relevant clinical, anamnestic, and radiological information necessary for the preparation of the histopathological diagnosis.

## 3. Specimen Fixation

Before sampling, the adequate fixation of the radical cystectomy (RC) specimen is recommended, either in the operating room or in the laboratory. This can be achieved through one of the following methods:

- Bladder cavity distension with an injection of 150–250 mL of buffered formalin (e.g., using a large-bore needle through the bladder dome or a Foley catheter through the urethra), followed by the clamping of the distal urethra. This approach ensures the proper fixation of the mucosa and any tumors within the bladder lumen while also promoting bladder wall distension for better macroscopic evaluation [1,2,3,4]. The specimen is then immersed overnight in an adequately sized container filled with formalin [3].
- Before immersion in a container with an adequate volume of formalin, the bladder should be opened anteriorly from the urethra to the bladder dome to ensure optimal fixation in an adequate volume of formalin [2].

A recent study by Griffin et al., based on an international survey of 212 pathologists, indicates that the latter method is the most commonly used in laboratory practice [5]. It was also adopted in a recent phase Ib study investigating the intravesical or direct tumor injection of atezolizumab in patients with bladder cancer prior to RC [6]. Currently, no statistically significant data suggest discrepancies in antigen preservation or nucleic acid quality between the two fixation methods. Recent multidisciplinary recommendations from the Brazilian Society of Pathology, the Brazilian Society of Urology, and the Brazilian Society of Clinical Oncology for the handling and reporting of bladder epithelial tumors also provide guidance on cold ischemia time (10 min to a maximum of 60 min), fixation duration (24–48 h), fixative characteristics (10% neutral buffered formalin, pH 6.9–7.1), and the fixative-to-tissue ratio (5:1 to 10:1) [1].

The next step involves inking the margin closest to any tumor visible within the perivesical adipose tissue or the entire specimen. Varma et al. [4] discourage the latter due to the risk of ink penetrating the perivesical adipose tissue and obscuring potentially present lymph nodes. If the specimen is sent fresh to the pathology laboratory, Compérat et al. recommend inking macroscopically visible lesions, such as areas of retracted or flattened mucosa (consistent with scars from previous TURB) or velvety reddish areas (suggestive of CIS), to ensure their identification after fixation [7].

## 4. Specimen Description

**Figure 2:**
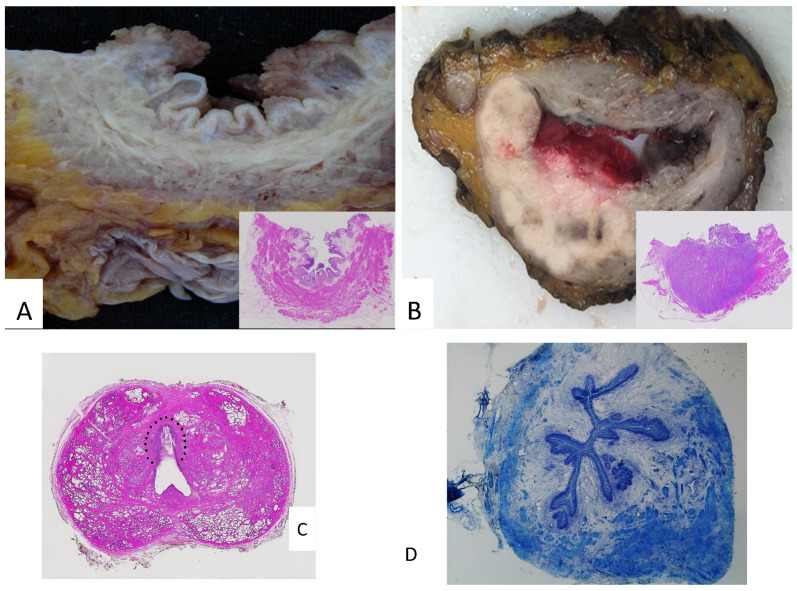
(**A**) Papillary lesions of the bladder: macroscopy and microscopy (inset). (**B**) A whole-mount bladder section featuring a solid neoplasm, which invades the perivesical adipose tissue: macroscopy and microscopy (inset). (**C**) A whole-mount prostate section featuring a urothelial carcinoma in the prostatic urethra (within the dotted line). (**D**) Frozen section of ureteral margin (Toluidine blu stain).

The macroscopic description of the specimen represents a fundamental step, especially since sampling and reporting are often carried out by different providers (in academic institutions, sampling may be often performed by pathology residents or fellows or, in other countries, pathologist assistants). For this reason, it is considered good practice to adequately document the contents of individual histological cassettes in relation to their anatomical location and lesion type [8]. Attaching photographic documentation and/or a diagram of the specimen may be useful [9]. Examining the specimen fresh (i.e., before formalin fixation) offers several advantages. In addition to allowing the evaluation of the in vivo color of the lesions, it enables proper fixation according to one of the previously described methods and facilitates the palpation of the specimen to identify areas of increased consistency that may not be visible to the naked eye [8]. A thorough macroscopic description includes the following:

- The dimensions of the bladder and any other removed organs do not have a universally accepted recommendation. This is because the clinical utility of such measurements is limited, along with the inherent differences in specimen size before and after formalin fixation [1,4,5,6,7,8,9,10]. Current EAU guidelines recommend that, in female cystectomy specimens, the length of the urethral segment removed en bloc with the bladder should be checked, preferably by a urological surgeon [11].
- A description of the internal bladder surface (Figure 2A,B) should be carried out: maximum tumor size, location, deepest invasion, and macroscopic appearance (flat, papillary, solid nodular, polypoid, or ulcerated), along with the status of the remaining mucosa and surgical margins. In addition to the maximum tumor dimension, it is advisable to report additional tumor measurements, as tumor diameter is a predictor of recurrence and disease-specific survival [12].

- The tumor location is particularly relevant when it is in the bladder dome, especially in the absence of a previous TUR histological examination, as differential diagnosis with urachal carcinoma is required. The latter has specific sampling and staging protocols [13].
- The presence or absence of the macroscopic invasion of perivesical adipose tissue or the serosa (qualifying the tumor as pT3b), as well as any lymph nodes or tumor deposits in perivesical adipose tissue, which should be carefully examined [4]. Suspected perivesical adipose tissue invasion by urothelial carcinoma should be distinguished microscopically from other conditions (e.g., peritumoral fibrosis) [13]. At this stage, inking the margin closest to the tumor is recommended [10]. Furthermore, perivesical fat should be carefully examined for lymph nodes or tumor deposits, which should be sampled accordingly [10].
- The description of the internal surface of the ureteral stumps, after longitudinal opening with scissors [4].
- The iliac–obturator lymph nodes removed concurrently. The lymphadenectomy specimen should be measured or, alternatively, weighed, then palpated and examined to isolate lymph nodes within the fibroadipose tissue [10]. The number and characteristics of the lymph nodes (e.g., the presence of macroscopic metastasis) should be reported. The lymph node diameter should be recorded if it cannot be determined on a histological slide [10].

## 5. Before Specimen Sampling

As mentioned above, providing accurate clinical information is of pivotal importance when handling RC specimens. Before sampling the specimen, the pathologist should be aware of the indication for surgery, tumor location, the number of prior TURs, any previous neoadjuvant therapy, and radiological findings regarding a possible extravesical extension or the presence of prostatic carcinoma [4]. When the tumor is not macroscopically visible (after a re-TUR or preoperative oncologic treatment), the pathologist’s attention, as well as the extent of sampling, should be guided by relevant clinical and anamnestic data, pre-treatment cystoscopic and radiological findings, and mucosal ulcerations or any other mucosal lesion. In the setting of post-neoadjuvant chemotherapy, the extensive sampling of the bladder is recommended, particularly in the clinically documented tumor site [2,4,14,15].

## 6. Specimen Sampling

After adequate fixation, the oriented bladder specimen should be entirely and transversely sectioned at 5 mm intervals from the bladder neck to the dome, allowing the slices to be better correlated with cross-sectional imaging from CT scans or MRI [2,14]. Each section should be individually examined to identify additional tumor foci, other lesions, or residual tumor in the post-neoadjuvant setting.

Histological sections should be obtained from the following:

- Tumor (if macroscopically evident): At least one section per centimeter of tumor, with the appropriate documentation of the macroscopically identified extent within the bladder wall for accurate pathologic staging [16]. This sampling aims to assess the tumor grade and histotype thoroughly. Some authors also recommend sampling apparently normal perilesional tissue with a margin of at least 1 cm [8].
- Seemingly normal mucosa from different bladder wall regions to detect occult multifocal carcinoma and/or urothelial carcinoma in situ (CIS). However, the extensive random sampling of macroscopically normal tissue is not recommended, as CIS identification in the bladder generally has limited clinical utility [4].

Even in the absence of a macroscopically identifiable tumor, whole-organ embedding is not recommended, as it does not provide a significant advantage in detecting histological prognostic parameters [17]. Griffin et al. [5] suggest that in cases where the tumor is not identifiable in the sampled material, it may be useful to consider step sections as an alternative to additional sampling. Aron et al. [8] recommend that, in the absence of macroscopically appreciable lesions, tissue samples should be taken from multiple bladder regions (the anterior wall, posterior wall, trigone, dome, right and left lateral walls, ureteral orifices, and urethral margin) for a total of 15 or more samples.

- Ureteral and urethral surgical resection margins should be sampled to identify any in situ or invasive tumors that are not macroscopically evident. Varma et al. recommend sampling the prostatic urethral margin of RC specimens with a slightly thicker section, as the distal prostatic urethra tends to retract after surgical resection and formalin fixation [4]. Both ureteral and urethral margins are obtained by shaving unless they have already been evaluated by frozen sections [9].

Current CAP guidelines recommend submitting one section for each ureteral margin and one for the urethral margin, with additional sections if a longer ureteral segment is present [18].

The EAU guidelines emphasize the importance of thoroughly documenting the radial, prostatic, ureteral, urethral, peritoneal fat, uterine, and vaginal vault margins [11].

- Associated organs in the RC surgical specimen (see above), regardless of the presence of a macroscopically detectable tumor. This aims to rule out the microscopic extension of bladder cancer and/or other primary tumors in adjacent organs, as well as to accurately determine the pathological stage [19].

In the case of the prostate, gland sampling should include both the areas adjacent to the bladder, near the bladder neck, to identify the direct invasion of bladder cancer into the prostate (pT4) and the periurethral region to detect the stromal invasion of the prostate by urothelial carcinoma. The latter may extend into the urethra or submucosal prostatic ducts, representing a synchronous urethral carcinoma, classified as pT2 if confined to the prostate gland [16]. Varma et al. recommend not sampling the bladder–prostate interface in cases where the tumor is resected from the bladder dome and no tumor is present at the bladder base [4].

The seminal vesicles and vas deferens should also be sampled, with particular attention to these structures when they are in proximity to the bladder tumor [9]. The current CAP guidelines recommend submitting sections from the prostatic urethra, including at the margin and with the surrounding prostatic parenchyma, as well as representative sections of the peripheral zone, central zone, and seminal vesicles [18]. In females, when the surgical specimen includes the uterus, the organ should be transversely sectioned to assess the potential invasion of the anterior uterine wall by a tumor located in the posterior bladder wall. If a macroscopically identifiable tumor is present near the vaginal margin, this margin should also be examined and sampled [4].

- Iliac–obturator lymph nodes: while a single section is sufficient for each lymph node with a macroscopically detectable metastasis, all macroscopically negative lymph nodes should be entirely submitted, as lymph node involvement may be microscopic and is used as an indication for adjuvant therapy [1,9,18].

In the absence of a clear indication regarding the minimum number of lymph nodes to be submitted for pathological evaluation, Aron et al. suggest that if fewer than three lymph nodes are macroscopically identified, the entire remaining fibroadipose tissue in each packet should be submitted [8]. This approach is particularly useful when lymph nodes may not be macroscopically identifiable due to fatty replacement [11].

## 7. Handling the Post-Chemotherapy Bladder

This approach is particularly relevant because tumor involvement is not infrequently found in perivesical adipose tissue, or as a perineural invasion or vascular emboli, even in the absence of a visible tumor on the mucosal surface or in the lamina propria [1]. However, opinions on this matter remain divided [20]. In a recent study, Saunders et al. [21] conducted a Qualtrics survey of 55 pathologists and pathology assistants, concluding that the prevailing gross examination practice consists of obtaining one section per centimeter (when the tumor is macroscopically identifiable) and extensively sampling the entire tumor or ulcer bed, along with radiology-guided sections (when the tumor is not macroscopically identifiable). The authors emphasize that the vast majority of pathologists are willing to obtain additional sections before signing out the case as ypT0. In such instances, they recommend documenting histologic changes observed in the previously documented tumor site (e.g., granulomas, histiocytes, hemosiderin/siderophages, and post-TUR giant cell reaction) rather than obtaining additional random sections. These conclusions are supported by findings from a previous study by the same authors, reporting that when NAC and non-NAC cases are sampled with a similar total number of blocks (approximately 15–22), adding extra random sections results in minimal upstaging (<2%) [22].

Assessing tumor response in BC patients undergoing NAC followed by RC is of pivotal importance for predicting survival outcomes. A meta-analysis demonstrated that patients achieving a pathologic complete response (pCR) after NAC had significantly better overall survival (OS) and recurrence-free survival (RFS) compared to those without pCR. Accordingly, a case–control matching study found that patients who received NAC before RC had a higher pCR rate (31% vs. 12%) and improved 5-year OS (89%) compared to those undergoing RC alone [23,24].

Furthermore, a retrospective multi-institutional study revealed that among patients with advanced-stage disease (cT3 or 4), responders (<pT2) to NAC had a significant survival benefit over non-responders (≥pT2) and those who did not receive NAC [25].

These data highlight the importance of implementing a dedicated protocol for the handling and sampling of RC specimens after NAC in order to accurately report the extent of the pathological response for prognostic purposes.

## 8. Issue in Prostate Sampling

As mentioned above, in the staging of bladder carcinoma, an extravesical tumor that directly and transmurally invades the prostatic stroma is classified as T4a, whereas a urothelial carcinoma located in the prostatic urethra that invades the prostatic stroma is classified as pT2 [26]. The fact that the former has a worse prognosis than the latter is supported by extensive scientific evidence [27,28]. There is considerable variability in prostatic sampling protocols for radical cystoprostatectomy specimens [17,29,30]. Some institutions routinely submit the entire prostate for histological evaluation, aiming to identify not only the microscopic extension of bladder cancer into the prostatic stroma but also incidental prostate carcinomas. Recently, Yoo et al. compared two groups, each with a distinct prostate sampling protocol in radical cystoprostatectomy specimens. In the first group (the conventional sampling group), random prostate samples were collected in the absence of macroscopically detectable lesions, while in the second group (the complete sampling group), the prostate was sampled according to the radical prostatectomy protocol, i.e., entirely sectioned at 4 mm intervals with the prior inking of the resection margins [31]. Their results showed a statistically significant difference in both the involvement of prostatic ducts and acini by urothelial carcinoma in situ and the extension of urothelial carcinoma into the prostatic stroma, with the second group showing higher rates of both than the first group. This had a greater impact on the final staging of bladder cancer when the prostate was fully evaluated in radical cystoprostatectomy specimens compared to conventional sampling. Based on these findings, the authors conclude that, particularly when the tumor is located in the trigone area or concurrent CIS is reported in a previous biopsy, a complete analysis of the prostate in RCP specimens is suggested. Conversely, other experts do not consider an extensive sampling of the prostate necessary in patients without a clinical or radiological suspicion of prostate cancer, arguing that it would resemble an oncological screening and provide limited clinical utility in the context of a confirmed bladder neoplasm requiring at least radical surgical treatment [3,4,20,32]. Among institutions that perform complete prostate sampling, the “whole-mount” technique, which allows one to obtain a large-format histological view of hematoxylin and eosin-stained sections (slide size 7.5 cm by 5.0 cm), can be used as an alternative to regular standard histological sections (slide size 7.5 cm by 2.5 cm) [33].

The advantages of whole-mount sections in this setting include a better visualization of the bladder wall architecture and its relationships with adjacent organs, as well as an easier correlation of pathological findings with radiological images (Figure 2C) [15,34].

## 9. Frozen Sections

Intraoperative analysis using frozen sections can be employed to assess the status of ureteral and urethral margins, as margin positivity has been associated with a higher risk of recurrence (Figure 2D) [35]. However, the risk of local recurrence after cystectomy is relatively low, and an invasive tumor at the margin is generally found in high-risk patients, who are more likely to develop distant metastases rather than local recurrence, ultimately determining patient outcomes. Regarding urothelial carcinoma in situ (CIS), it is generally challenging to identify on frozen sections, particularly for pathologists not specialized in uropathology. Although CIS is associated with a higher recurrence rate in the upper urinary tract, its biological characteristics mean that negative resection margins do not exclude the presence of residual tumor [4]. Therefore, an intraoperative analysis of ureteral margins may not be justified in all cases [36]. On the other hand, the presence of CIS at the urethral margin has clinically significant implications for the construction of a neobladder. Hence, greater attention should be given to sampling and evaluating the urethral margin, both in an intraoperative assessment and in conventional sections [20].

## 10. Conclusions

The pathological evaluation of radical cystectomy specimens is a crucial step in the management of bladder cancer. Given the variability in clinical practices, a standardized approach is essential to optimize diagnostic accuracy and ensure comprehensive tumor staging. While international guidelines offer general recommendations, differences remain in fixation methods, sampling protocols, intraoperative margin assessments, and inter-laboratory practices. Moreover, variations in resource availability and the expertise of various pathologists can result in discrepancies in tumor staging and margin evaluation, highlighting the need for further standardization. This review emphasizes the importance of systematic specimen handling, particularly regarding key anatomical structures, margin evaluation, and lymph node assessment. Future efforts should concentrate on refining protocols through multidisciplinary collaboration among pathologists, urologists, and oncologists, integrating technological advancements such as artificial intelligence-assisted analysis and novel molecular markers to improve staging and prognostic assessment.

Challenges in standardizing radical cystectomy specimen handling continue due to inter-laboratory variability, resource inequalities, and complexities in protocol implementation. Standardization in pathological workflows will ultimately facilitate improved clinical decision making and enhance patient outcomes. Future multicentric studies that compare different fixation and sampling techniques could offer additional insights into the most effective methodologies for managing bladder cancer. Furthermore, incorporating molecular profiling into routine histopathological evaluations may enhance risk stratification and guide personalized treatment strategies.
